# Modulating Osteoclast Activity and Immune Responses with Ultra-Low-Dose Silver Nanoparticle-Loaded TiO_2_ Nanotubes for Osteoporotic Bone Regeneration

**DOI:** 10.3390/jfb16050162

**Published:** 2025-05-04

**Authors:** Zhen Wang, Penghui Xiang, Zhe Xu, Meiqi Gu, Rui Zhang, Yifei Li, Fei Xin, Chengla Yi

**Affiliations:** Department of Traumatic Surgery, Tongji Hospital, Tongji Medical College, Huazhong University of Science and Technology, Wuhan 430030, China; d202282250@hust.edu.cn (Z.W.);

**Keywords:** biomaterials, bone defects, osteoclast, inflammation, macrophages, autophagy

## Abstract

Introduction: Osteoporosis results from the dysregulation of osteoclast activation mechanisms. The subsequent inflammation in osteoporotic environments further hampers bone healing and impedes osseointegration. Therefore, developing treatments that can modulate osteoclast activity and regulate immune responses is essential for effectively treating osteoporotic bone defects. Methods: In this study, silver nanoparticle-decorated TiO_2_ nanotubes (Ag@TiO_2_-NTs) were synthesized through an electrochemical anodization technique for surface modification. The morphology and elemental composition of the Ag@TiO_2_-NTs structures were characterized using scanning electron microscopy (SEM) and related methods. Subsequently, a series of in vitro and in vivo experiments were conducted to investigate the regenerative potential of Ag@TiO_2_-NTs in osteoporotic bone defects. In vitro assays focused on evaluating cell viability and osteoclast function, while in vivo assessments employed osteoporotic rat models to monitor bone healing via histological examination and micro-computed tomography (micro-CT) imaging. Results: Our results demonstrated that Ag@TiO_2_, through the controlled release of trace amounts of silver ions, significantly suppressed osteoclast activity and consequently alleviated bone resorption under osteoporotic conditions. In addition, Ag@TiO_2_-NTs facilitated the polarization of macrophages toward the M2 phenotype. These biological effects were associated with the stimulation of autophagy, a fundamental mechanism involved in cellular repair. Moreover, the activation of autophagy contributed to the suppression of RANKL-induced NF-κB signaling, a pathway essential for the regulation of bone metabolism Conclusion: These results suggest that this surface modification strategy has the potential to be an ideal implant biomaterial for treating osteoporotic bone defects and a promising strategy for future implant surgeries.

## 1. Introduction

Osteoporosis is one of the most common diseases in aging people, characterized by significant bone loss and increased bone fragility [1,2,3]. With the aging population, osteoporosis has become a global health issue [4,5]. Dysregulated osteoclast generation is a major cause of osteoporosis [6,7]. Under osteoporotic conditions, excessive osteoclast activity leads to delayed bone defect healing [8]. On the other hand, the macrophages activated in osteoporotic conditions secrete a range of inflammatory cytokines, disrupting the local bone immune balance and further impairing implant osseointegration [9,10]. Therefore, the development of biomaterials that regulate osteoclast activity and macrophage-related immune responses is critical for treating osteoporotic bone defects.

Osteoclasts are bone-specific multinucleated cells originating from the monocyte–macrophage hematopoietic lineage, and they play a central role in bone resorption, being essential for bone remodeling, repair, and mineral homeostasis [11]. Osteoclasts and macrophages are key regulators of bone metabolism. Osteoclasts, originating from the monocyte/macrophage lineage, are multinucleated cells primarily involved in bone resorption [12]. The interplay between osteoblasts and osteoclasts is essential for preserving bone homeostasis and regulating bone remodeling. While osteoblasts drive new bone formation, osteoclasts are responsible for its resorption, and their coordinated activity maintains the dynamic balance of bone tissue renewal [13]. Macrophages participate in regulating bone metabolism through their diverse functions. Studies have shown that the polarization state of macrophages can influence the generation and function of osteoclasts. M1-type macrophages typically promote the formation of osteoclasts, whereas M2-type macrophages facilitate the osteoblastic process [14]. During bone remodeling, osteoclasts dissolve the bone matrix by secreting enzymes and acidic substances, while macrophages clear residual bone resorption debris through phagocytosis and release signaling molecules to regulate osteoblast activity [15]. The receptor activator of nuclear factor κB ligand (RANKL), by binding to its receptor RANK, plays a pivotal role in regulating osteoclast differentiation and survival [16]. Moreover, osteoclastogenesis relies on the transcriptional activities of NFATc1, cathepsin K (CTSK), and c-Fos, all of which are triggered by the nuclear factor-κB (NF-κB) signaling pathway, partially attributable to RANK recruitment [17,18]. Hence, osteoclast activity can be suppressed by inhibiting the downstream signaling pathways induced by RANKL [19]. Targeting RANKL-induced osteoclastogenesis to modulate excessive bone resorption may be an effective strategy for treating osteoporotic bone defects.

Titanium dioxide nanotubes (TiO_2_-NTs) have demonstrated significant potential in enhancing bone formation and regulating immune activity [20,21]. The strategies applied in are considered novel surface strategies for controlled drug delivery systems. Silver nanoparticles (AgNPs) have garnered particular attention due to their well-known antimicrobial properties and potential osteoinductive effects [22,23]. Additionally, AgNPs have been shown to modulate the proliferation and differentiation of the mesenchymal stem cells (MSCs) involved in bone regeneration [23]. Interestingly, recent studies have reported that biomaterials loaded with AgNPs, which release ultra-low doses of Ag ions (Ag^+^), can enhance cell adhesion, diffusion, proliferation, reactive oxygen species (ROS) clearance, and new bone formation through their unique controlled release and topographic features [24,25,26,27]. However, few reports have addressed the role of AgNPs in osteoclast differentiation.

Despite extensive research, only a few biomaterials have successfully been translated into clinical practice due to the complexity of the immune microenvironment network. Host immune responses can lead to the formation of surrounding inflammatory fibrous tissue around some implants, resulting in the loss of osseointegration efficiency [28]. Macrophages play a key role in the early host immune response by recognizing foreign materials and differentiating into two major phenotypes: pro-inflammatory M1 and wound-healing M2 phenotypes [29,30]. Recent studies have shown that the presence of chronic M1 macrophages at the bone–implant interface inhibits new bone formation and promotes the inflammatory encapsulation of the implant [31,32]. Furthermore, a higher ratio of M1/M2 macrophages has been found to be strongly correlated with the failure of joint replacement surgeries [33]. Therefore, modulating the M1/M2 macrophage ratio and promoting macrophage polarization toward the M2 phenotype may be crucial for inhibiting local adverse inflammation, achieving an appropriate immune response, and promoting tissue regeneration [34,35]. 

Autophagy is a highly conserved and ubiquitous cellular activity that maintains homeostasis by degrading excess and damaged intracellular components [36]. In recent years, increasing evidence has shown a close relationship between autophagy and bone metabolism, garnering significant attention [37]. Studies have indicated that calcium and vitamin D3 can induce autophagy, promoting osteoblastic differentiation [38,39]. Ha et al. discovered that silica nanoparticles can enhance osteoblast differentiation by inducing autophagy [40]. Song et al. demonstrated that different surface morphologies of titanium implants can trigger autophagic responses in various cell lines via the rapamycin target of the mTOR signaling pathway [41]. Thus, leveraging biomaterials, especially nanomaterials, to trigger autophagy is an attractive research direction, as it may offer therapeutic properties through the molecular/drug loading of the materials or their inherent characteristics (e.g., shape, roughness, and surface chemistry, etc.) [42,43]. This holds significant clinical implications for discovering autophagy-related signals, pathways, mechanisms, and therapies in bone diseases.

In this study, we aimed to explore the potential of Ag@TiO_2_-NTs as a therapeutic material for enhancing bone regeneration in osteoporotic conditions. Specifically, we fabricated Ag@TiO_2_-NTs through an electrochemical anodization process to enable the controlled release of ultra-low doses of silver ions (Ag^+^). The hypothesis driving this research was that the controlled release of silver ions would not only inhibit osteoclastogenesis, but also modulate immune responses by promoting M2 macrophage polarization, thereby facilitating bone regeneration in osteoporotic bone defects.

The primary objective of this study was to investigate the immunomodulatory effects of Ag@TiO_2_-NTs on RANKL-induced osteoclastogenesis and macrophage polarization in vitro. By understanding how Ag@TiO_2_-NTs influences these cellular processes, we aimed to shed light on its mechanisms of action at the cellular level. Additionally, we sought to evaluate the impact of Ag@TiO_2_-NTs on the regeneration of osteoporotic bone defects in ovariectomized (OVX) rats, which is a well-established model for postmenopausal osteoporosis. Our findings may provide valuable insights into the therapeutic potential of Ag@TiO_2_-NTs for treating osteoporotic bone defects, offering a novel approach to stimulating bone regeneration through immune modulation and controlled silver ion release.

## 2. Materials and Methods

**Figure 1:**
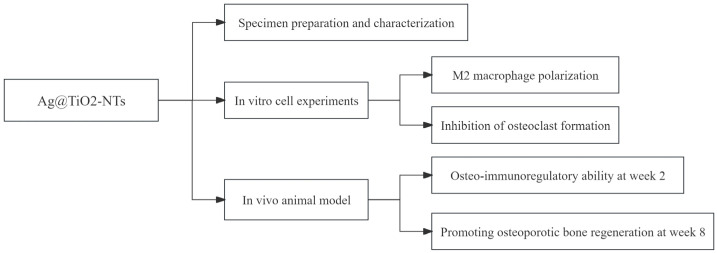
Flow sheet demonstrating the study design.

The study design is illustrated in Figure 1.

### 2.1. Ag@TiO_2_-NTs Preparation

The materials and titanium sheets (10 mm × 10 mm × 1 mm, Thermo Fisher Scientific Inc.,Waltham, MA, USA) were sequentially polished with 400#, 1000#, 1500#, and 2000# sandpaper to remove surface scratches and oxide layers. The polished sheets were sonicated in acetone, anhydrous ethanol, and distilled water for 5 min each to remove impurities. Finally, they were dried with nitrogen for later use. A polishing solution was prepared by mixing 200 mL distilled water, 200 mL nitric acid, 50 mL hydrofluoric acid, and 50 mL distilled water. The pretreated titanium sheets were immersed in the solution, sonicated for 1 min and 26 s, then rinsed twice with distilled water for 5 min. The polished sheets were dried with nitrogen. Graphite and pretreated titanium sheets served as the cathode and anode, respectively, with a 1 cm distance between them. The electrolyte bath was maintained at 17–18 °C under stirring. A voltage ramp from 0 V to 5 V (0.1 V/s) was applied for 3 min, followed by an increase to 60 V (0.1 V/s), and held for 30 min. After anodization, the titanium sheets turned yellow, indicating the formation of an oxide layer. The sheets were rinsed with distilled water and dried with nitrogen. The samples were then heated at 500 °C for 3 h to crystallize the oxide layer. After cooling, the residual electrolyte was removed by washing with a water–ethanol mixture (1:1) and sonicated until no white precipitates appeared. Finally, the samples were dried with nitrogen. The electrolyte was prepared by dissolving 1.6374 g NH_4_F, 15 mL distilled water, 15 mL methanol, and 270 mL ethylene glycol in a plastic bottle, followed by 3 h of stirring. The TiO_2_-NTs were immersed in 20 mL of 0.1 M AgNO_3_ solution for 15 min and irradiated with UV light for 30 min to obtain Ag-loaded TiO2-NTs (Ag@TiO2-NTs). Our immersion method was designed to ensure uniform AgNP distribution by maintaining an adequate liquid phase for redox reactions during UV irradiation, as supported by previous studies [22,44]. 

### 2.2. Characterization

The surface morphology of the samples (Ti, TiO_2_-NTs, and Ag@TiO_2_-NTs) was examined using scanning electron microscopy (SEM, FEI Nova400 Nano, Waltham, MA, USA). Energy-dispersive X-ray spectroscopy (EDS; Tecnai G20, FEI, Waltham, MA, USA) was used to analyze the chemical composition. The hydrophilicity of the samples was measured using a contact angle goniometer (DSA1, Kruss, Hamburg, Germany).

In this study, the size of AgNPs was estimated to be ~20 nm based on the synthesis protocol adapted from prior methodologies [22,44]. For the release of Ag^+^ ions, 0.01 mol/L PBS (pH 7.4) was used to immerse the Ag@TiO_2_-NTs at various time points. After incubation, the Ag^+^ concentration of the PBS solution was analyzed using flame atomic absorption spectroscopy (TAS-990, Beijing Pusix, China).

### 2.3. Cell Culture and Proliferation

RAW 264.7 macrophages and MC3T3-E1 preosteoblasts (Chinese Academy of Sciences, Shanghai, China) were cultured in DMEM (Gibco, Grand Island, NY, USA) with 10% FBS and 1% penicillin/streptomycin in a 5% CO_2_ atmosphere at 37 °C. Cell proliferation was assessed using a CCK-8 assay (Beyotime, Shanghai, China) after 24, 48, and 72 h. Absorbance was measured at 450 nm using a microplate reader (Nikon, Tokyo, Japan).

### 2.4. Cell Inflammatory Response

RAW 264.7 cells were polarized to M1 macrophages using LPS. RAW 264.7 is a widely recognized murine macrophage cell line extensively utilized to study osteoclastic differentiation due to its ability to differentiate into functional osteoclasts under RANKL stimulation [45,46]. The inflammatory response was assessed by culturing RAW 264.7 cells on Ti, TiO_2_-NTs, and Ag@TiO_2_-NTs (1 × 1 cm^2^) for 24 h. Supernatants were collected for ELISA analysis. The expression levels of pro-inflammatory (TNF-α, iNOS) and anti-inflammatory (TGF-β, Arginase1) biomarkers were observed via confocal microscopy (Olympus, Tokyo, Japan). ELISA kits (R&D Systems, Minneapolis, MN, USA) were used to quantify TNF-α and TGF-β in the supernatants.

### 2.5. Macrophage–Preosteoblast Co-Culture Assay

RAW 264.7 cell-conditioned medium (CM) was collected after culturing for 24 h on different samples (Ti, TiO_2_-NTs, and Ag@TiO_2_-NTs) and co-cultured with MC3T3-E1 preosteoblasts for 72 h. Osteogenic differentiation was assessed via qPCR for ALP, OCN, OPN, and RUNX2 gene expression.

### 2.6. Osteoclastogenesis Assay

RAW 264.7 cells and bone marrow-derived macrophages (BMMs) were cultured in DMEM with 10% FBS and 1% penicillin/streptomycin, and induced with M-CSF (30 ng/mL) and RANKL (50 ng/mL). Osteoclastogenesis was assessed after 24 and 72 h for RAW 264.7 cells and 3 and 7 days for BMMs. TRAP staining was used to test osteoclast differentiation, and F-actin immunofluorescence staining was used to identify mature osteoclasts.

### 2.7. In Vivo Animal Model

In this study, six ovariectomized 6–8-week-old female Sprague Dawley rats per group were used to establish an osteoporosis model for the in vivo experiments. A 2.0 × 6.0 mm defect was created in the iliac bone after 12 weeks of ovariectomy, and Ti, TiO_2_-NTs, and Ag@TiO_2_-NTs were implanted. The defects were randomly divided into three groups: Ti, TiO_2_-NTs, and Ag@TiO_2_-NTs. The rats were euthanized at 2 and 8 weeks post surgery for further analysis, including micro-CT and histological examination.

### 2.8. Micro-CT and Histological Analysis

Micro-CT scans were performed using a Bruker MicroCT Skyscan 1176 system to evaluate the bone structure and new bone formation. For histological analysis, iliac bones were decalcified in 10% EDTA, embedded in paraffin, and sectioned at a 4 μm thickness. Hematoxylin and eosin (H&E), Masson’s trichrome, and TRAP staining were performed, along with immunohistochemistry for macrophage markers (CD68, TNF-α, and TGF-β).

### 2.9. Gene and Protein Expression

Gene expression was quantified via RT-qPCR using a PrimeScript RT Reagent Kit (Takara, Kusatsu, Japan). The primers used for reverse transcription quantitative PCR in this study are shown in Table A1. Protein expression was analyzed via Western blotting with primary antibodies against LC3, p-IκBα, p65, p-p65, and β-actin, followed by incubation with secondary antibodies.

### 2.10. Statistical Analysis

GraphPad Prism software, version 9.0 was used for statistical analyses. All experiments were repeated three times, and the data are presented as mean ± standard deviation. Statistical significance was determined via one-way ANOVA with the Student–Newman–Keuls post hoc test. In this study, 6 rats per group were used for the in vivo experiments, and the sample size was statistically justified based on effect sizes from prior studies and in compliance with institutional animal ethics guidelines [47,48].

Differences were considered significant at *p* < 0.05 (* *p* < 0.05, ** *p* < 0.01, *** *p* < 0.001, and **** *p* < 0.0001).

## 3. Results

### 3.1. Surface Characterization, Ag^+^ Release, and Proliferation

**Figure 2:**
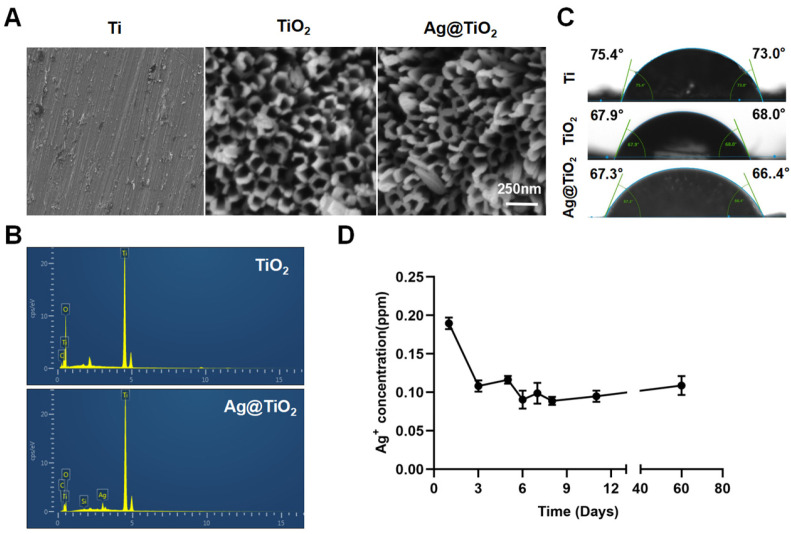
Surface characterization and Ag+ release of implant. (**A**) Representative scanning electron microscopy (SEM) images depict the surface morphology of the different sample groups, highlighting the distinct structural characteristics of Ti, TiO_2_-NTs, and Ag@TiO_2_-NTs substrates. (**B**) Energy-dispersive X-ray (EDX) spectra for TiO_2_-NTs and Ag@TiO_2_-NTs confirm the successful incorporation of silver within the Ag@TiO_2_-NTs composite. (**C**) Measurements of water contact angles for Ti, TiO_2_-NTs, and Ag@TiO_2_-NTs reveal notable differences in surface wettability among the materials. (**D**) The release profile of Ag⁺ ions at various intervals (days 1, 3, 5, 6, 7, 8, 11, and 2 months) demonstrates a controlled and sustained release behavior over time.

SEM images showed that the ordered nanotube arrays on the titanium surface, with a diameter of approximately 120 nm, were fabricated via conventional anodization (Figure 2A). In this study, Ag^+^ was reduced using high-pressure Hg lamps to prepare AgNPs. This method does not require reducing agents or heat treatment. Figure 2B shows the corresponding EDX spectra, which indicate the chemical composition of the surface of the samples. Compared to TiO_2_-NTs, Ag@TiO_2_-NTs exhibit the presence of Ag, indirectly confirming the successful loading of AgNPs onto the TiO_2_-NT layer. Figure 2C presents the water contact angles of Ti, TiO_2_-NTs, and Ag@TiO_2_-NTs, which were 75.4° (left) and 73.0° (right), 67.9° (left) and 68.0° (right), and 67.3° (left) and 66.4° (right), respectively. This shows that the nanotube structures on the Ti surface increase the hydrophilicity of the material. Additionally, the Ag^+^ release kinetics are shown in Figure 2D, where the concentration of Ag^+^ was analyzed using flame atomic absorption spectroscopy at day 1, 3, 5, 6, 7, 8, 11, and month 2. The release curve indicates that, except for the first day, the concentrations of Ag^+^ were relatively stable, with the highest concentration on day one, and the levels remaining within a low range thereafter. After two months of immersion, the Ag^+^ concentration reached a saturation point in PBS solution and did not significantly increase with a prolonged immersion time.

**Figure A1:**
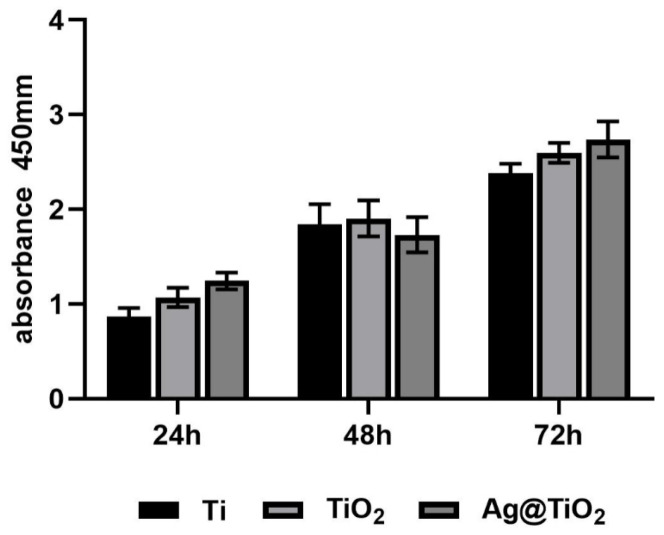
CCK-8 assays for MC3T3-E1 of different groups at 24 h, 48 h, and 72 h.

To eliminate the potential toxicity of AgNPs on cell proliferation, ultra-low concentrations of AgNPs (releasing about 0.2 ppm in the first 24 h) were loaded onto TiO_2_-NTs (Figure 2D). CCK-8 assays (Figure A1) showed no significant differences between the groups. In summary, Ag@TiO_2_-NTs with ultra-low-dose AgNPs exhibited no significant cytotoxicity.

### 3.2. Osteo-Immunoregulatory Ability In Vitro

**Figure 3:**
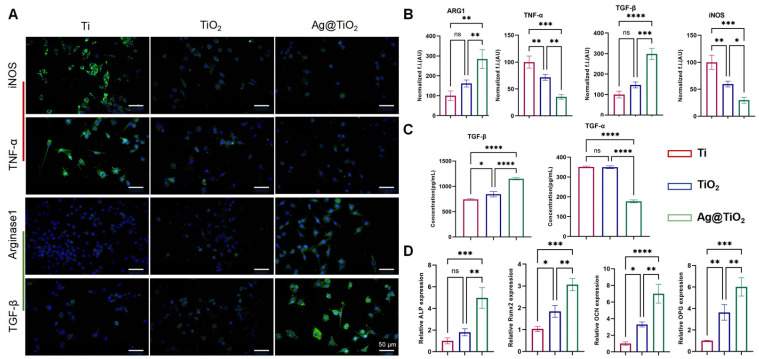
Osteo-immunoregulatory ability in vitro. (**A**) Representative fluorescence images showing the expression of iNOS, TGF-α, Arginase 1, and TGF-β in different groups, illustrating the immunomodulatory effects on macrophage polarization and related cytokine production. (**B**) Quantitative analysis of iNOS, TGF-α, Arginase 1, and TGF-β levels in different groups, providing a comparison of their expression to assess the immune response and modulation by the treatments. (**C**) ELISA analysis of TGF-α and TGF-β levels in the groups, offering further quantification of these key cytokines involved in the osteo-immunoregulatory process. (**D**) mRNA expression of osteogenic genes ALP, Runx2, OCN, and OPN, evaluating the potential of the materials to promote osteogenesis and bone healing by influencing the expression of critical markers in osteoblast differentiation and function. Significance levels: * *p* < 0.05, ** *p* < 0.01, *** *p* < 0.001, **** *p* < 0.0001; ns = not significant. Error bars represent SEM (standard error of the mean).

The LPS-treated macrophages were seeded onto Ti, TiO_2_-NTs, and Ag@TiO_2_-NT surfaces, and after two days of culture, the expression of M1 (iNOS and TNF-α) and M2 (Arginase1 and TGF-β) markers was analyzed via immunofluorescence staining and ELISA. As shown in Figure 3A,B, compared to Ti and TiO_2_-NTs, the fluorescence signal of iNOS and TGF-α in the Ag@TiO_2_-NTs group was much lower, while Arginase1 and TGF-β followed an opposite trend. Furthermore, the ELISA of TGF-α and TGF-β showed a similar trend (Figure 3C). We used a traditional co-culture method with macrophage-conditioned medium (CM) to treat MC3T3-E1 to investigate the effects of Ag@TiO_2_-NTs on osteogenic gene expression in MC3T3-E1 cells. The results showed that the CM from the Ag@TiO_2_-NTs group significantly promoted the expression of osteogenic genes ALP, Runx2, OCN, and OPN (Figure 3D).

### 3.3. Ag@TiO_2_-NTs Inhibited RANKL-Induced Osteoclastogenesis In Vitro

**Figure 4:**
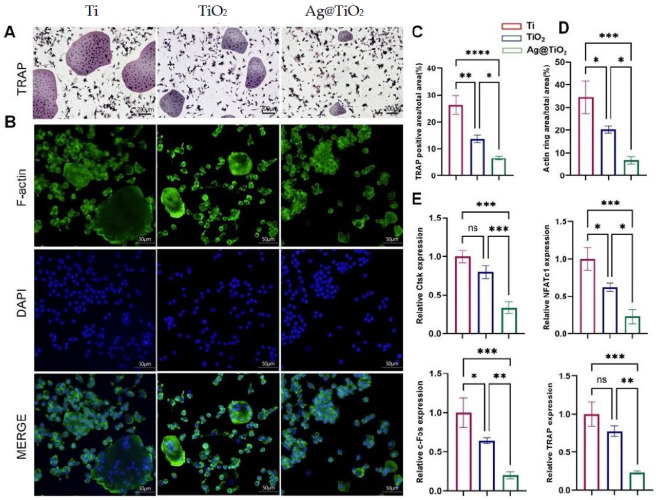
Ag@TiO_2_-NTs inhibited RANKL-induced osteoclastogenesis in vitro. (**A**) Representative TRAP staining images of osteoclasts in different groups, highlighting the effect of Ag@TiO_2_-NTs on osteoclast differentiation. (**B**) Representative fluorescence images of F-actin in different groups, illustrating the cytoskeletal changes associated with osteoclast activity and differentiation. (**C**) Quantitative analysis of TRAP activity in different groups, providing a measure of osteoclast differentiation and function. (**D**) Quantitative analysis of F-actin in different groups, reflecting the impact of Ag@TiO_2_-NTs on the cytoskeletal organization of osteoclasts. (**E**) mRNA expression of osteoclast-specific genes NFATc1, c-Fos, TRAP, and Ctsk, assessing the molecular regulation of osteoclastogenesis and confirming the inhibitory effect of Ag@TiO_2_-NTs on osteoclast differentiation. Significance levels: * *p* < 0.05, ** *p* < 0.01, *** *p* < 0.001, **** *p* < 0.0001; ns = not significant. Error bars represent SEM (standard error of the mean).

To evaluate osteoclast differentiation, tartrate-resistant acid phosphatase (TRAP), a well-known marker for osteoclasts, staining was performed, and F-actin immunofluorescence staining was used to reveal mature osteoclast formation. The TRAP activity and F-actin formation of BMMs under RANKL and m-CSF induction were evaluated to assess the level of osteoclast differentiation. The inhibitory effects of Ti, TiO_2_-NTs, and Ag@TiO_2_-NTs on RANKL-induced osteoclast differentiation was assessed using TRAP and F-actin immunofluorescence staining (Figure 4A,B). Quantitative analysis revealed that Ag@TiO_2_-NTs significantly inhibited osteoclast differentiation and maturation compared to Ti and TiO_2_-NTs groups (Figure 4C,D). qRT-PCR analysis identified the downregulated expression of osteoclast-specific genes such as Ctsk, NFATc1, c-Fos, and TRAP in the Ag@TiO_2_-NTs group upon RANKL induction (Figure 4E).

### 3.4. Ag@TiO_2_-NTs Inhibited RANKL-Induced Osteoclastogenesis Through Modulation of Autophagy and NF-κB Pathways

**Figure 5:**
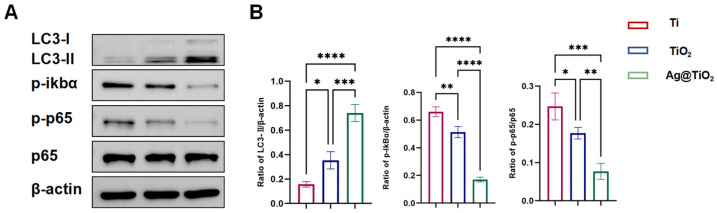
Ag@TiO_2_-NTs inhibited RANKL-induced osteoclastogenesis through modulation of autophagy and NF-κB pathways. (**A**) Western blot analysis of autophagy and NF-κB protein expression in different groups, showing the impact of Ag@TiO_2_-NTs on key signaling pathways involved in osteoclastogenesis. (**B**) Quantitative analysis of autophagy and NF-κB protein expression, providing a comparison of protein levels in the different groups to assess the modulation of these pathways by Ag@TiO_2_-NTs. Significance levels: * *p* < 0.05, ** *p* < 0.01, *** *p* < 0.001, **** *p* < 0.0001. Error bars represent SEM (standard error of the mean).

Autophagy serves as a fundamental cellular mechanism for maintaining homeostasis and regulating immune responses, partly by mitigating oxidative stress through the removal of reactive oxygen species (ROS). To investigate autophagy activity, the expression levels of key markers in RANKL-stimulated RAW 264.7 cells were assessed via Western blot analysis, focusing on LC3, a core protein associated with autophagosome formation. Quantitative analysis revealed that Ag@TiO_2_-NTs significantly enhanced autophagy in RANKL-induced RAW 264.7 cells compared to the Ti and TiO_2_-NTs groups (Figure 5A,B). The NF-κB signaling pathway is a classic pathway involved in osteoclast activation. Western blot analysis was performed to assess the inhibition of the NF-κB pathway in RANKL-induced osteoclasts. Quantitative analysis revealed that Ag@TiO_2_-NTs had a significantly stronger inhibitory effect on the NF-κB signaling pathway in RANKL-induced osteoclasts compared to the Ti and TiO_2_-NTs groups (Figure 5A,B). In summary, Ag@TiO_2_-NTs inhibited RANKL-induced osteoclastogenesis by enhancing autophagy and suppressing the NF-κB pathway.

### 3.5. Ag@TiO_2_-NTs Implant Stimulated Bone Regeneration in OVX Rats

**Figure 6:**
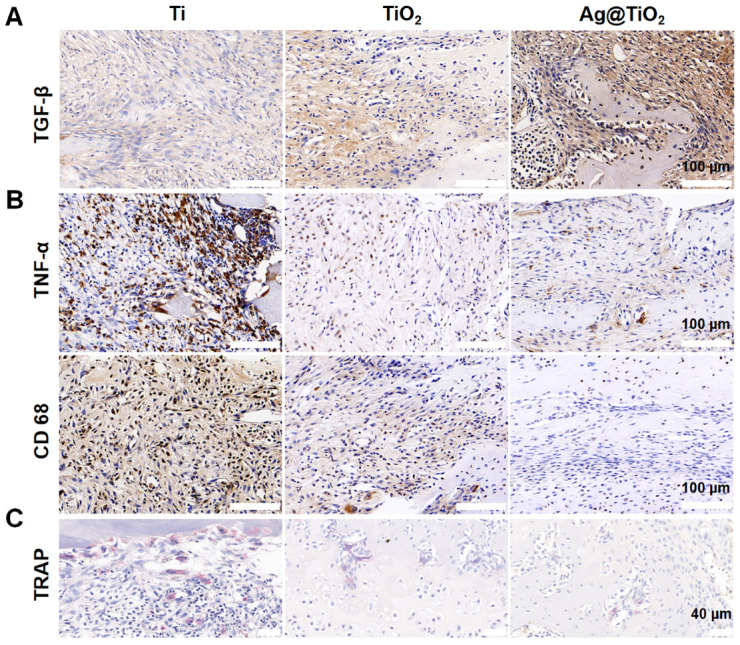
Ag@TiO_2_-NTs implant created an osteo-immune microenvironment in OVX rats. (**A**) Representative immunohistochemical staining of TGF-β in different groups after 2 weeks, illustrating the expression of this key cytokine involved in osteogenesis and inflammation. (**B**) Representative immunohistochemical staining of TNF-α and CD68 in different groups after 2 weeks, showing the inflammatory response and macrophage activity in the treated groups. (**C**) Representative images of TRAP staining in different groups after 2 weeks, highlighting the osteoclast differentiation and activity in response to the treatments.

**Figure A2:**
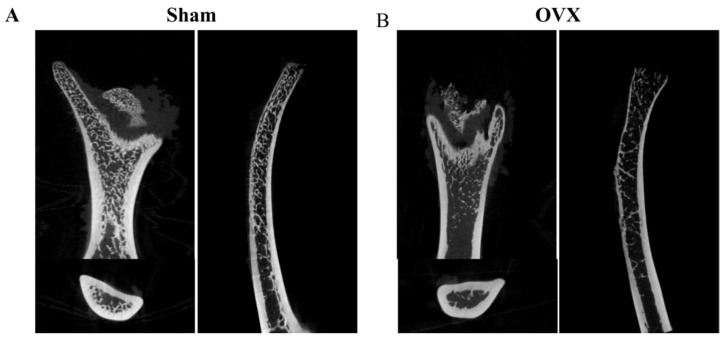
The osteoporotic model was established in OVX rats. (**A**) Representative μ-CT images in sham model. (**B**) Representative μ-CT images in OVX model.

We evaluated the effects of Ag@TiO_2_-NTs implants on in vivo bone immune microenvironment modulation, bone resorption inhibition, and osteoporotic bone defect regeneration. An osteoporotic model was established in OVX rats (Figure A2), and the implants were placed into the iliac bone defects to create the osteoporotic iliac bone defect model. After 2 weeks, the osteo-immune microenvironment was assessed through inflammatory biomarkers. Immunohistochemical staining showed higher expression of TGF-β around the Ag@TiO_2_-NTs implant group (Figure 6A), indicating more M2 macrophages. Simultaneously, lower levels of TNF-α and CD68 expression were detected around the Ag@TiO_2_-NTs implant group, suggesting reduced M1 polarization and macrophage numbers (Figure 6B). The decrease in TRAP indicated that the number of osteoclasts was the lowest in the Ag@TiO_2_-NTs implant group (Figure 6C).

**Figure 7:**
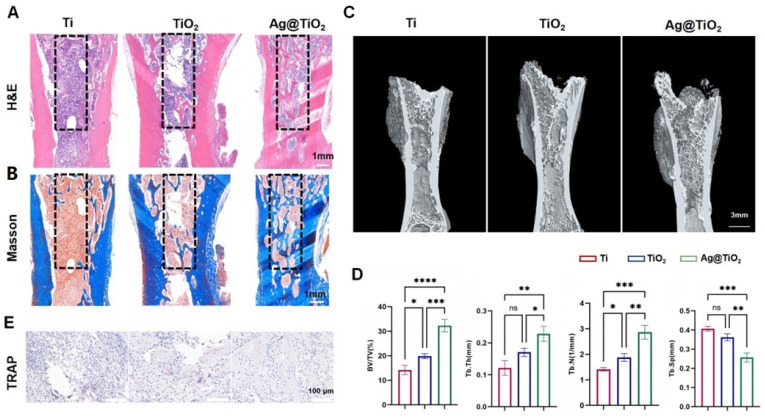
In vivo results at 8 weeks post iliac bone defect induction. (**A**) Representative H&E staining in different groups, providing histological details on tissue morphology and bone healing after treatment. (**B**) Representative Masson staining in different groups, assessing collagen deposition and the extent of bone matrix formation in the treated groups. (**C**) Representative μ-CT images in different groups, showing 3D reconstructions of bone regeneration within the iliac defects, highlighting differences in bone formation and healing. (**D**) Micro-CT parameters of bone regeneration within iliac defects in different groups, quantitatively evaluating bone volume, density, and other key structural aspects. (**E**) Representative images of TRAP staining in different groups, illustrating osteoclast differentiation and activity, which is critical for understanding bone resorption and healing dynamics. Significance levels: * *p* < 0.05, ** *p* < 0.01, *** *p* < 0.001, **** *p* < 0.0001; ns = not significant. Error bars represent SEM (standard error of the mean).

At 8 weeks post iliac bone defect induction, H&E and Masson staining indicated a small amount of new bone tissue in the defect of the Ti implant group (Figure 7A,B). The TiO_2_-NTs implant group showed a moderate amount of new bone tissue around the defect area, while large empty spaces still remained in the defect area. In contrast, the Ag@TiO_2_-NTs implant group exhibited uniformly distributed new bone tissue throughout the defect. Additionally, 3D images at week 8 showed more new regenerating bone in the Ag@TiO_2_-NTs implant group compared to the Ti and TiO_2_-NTs implant groups (Figure 7C). The percent bone volume (BV/TV)), trabecular thickness (Tb.Th), and trabecular number (Tb.N) in the Ag@TiO_2_-NTs implant group were significantly higher, and the trabecular separation (Tb.Sp) was significantly lower compared to the Ti and TiO_2_-NTs implant groups (Figure 7D). Moreover, the decrease in TRAP indicated that osteoclastogenesis around the Ag@TiO_2_-NTs implants was suppressed (Figure 7E).

## 4. Discussion

The dynamic equilibrium of the skeletal system relies on continuous remodeling orchestrated by osteoblasts and osteoclasts [49]. An imbalance between osteoblast-driven bone formation and osteoclast-mediated bone resorption results in bone loss and increases the risk of osteoporotic fractures, with osteoclast hyperactivation playing a central role in the underlying pathogenesis [7,50]. In this study, inhibiting osteoclast differentiation may be an effective strategy for treating osteoporosis. Furthermore, regulating macrophage M2 polarization to reduce inflammation could help create a favorable immune microenvironment around implants. In this study, we identified and clarified the role of Ag@TiO_2_-NTs with the controlled release of ultra-low-dose Ag^+^, which inhibits osteoclastogenesis and promotes the regeneration of osteoporotic bone defects by activating autophagy and suppressing RANKL-induced NF-κB signaling. Additionally, Ag@TiO_2_-NTs can promote M2 polarization of macrophages, thus modulating the immune microenvironment to facilitate osteogenesis.

This study successfully developed Ag@TiO_2_-NTs with the controlled release of ultra-low-dose Ag^+^ ions, using TiO_2_-NTs as a matrix. Notably, the AgNP size serves as an important factor of cytotoxic behavior. Chen et al. demonstrated that at around 20 nm, AgNPs had no statistical cytotoxicity in their experimental model [22,44]. Although some nanoparticles have been used in orthopedics, very few are suitable for treating osteoporosis. Over the recent decades, GNPs (gold nanoparticles) have been promising candidates for inhibiting osteoclast differentiation [51,52,53,54,55]. However, there are few reports on the effects of SNPs (silver nanoparticles) in inhibiting osteoclasts. Lee et al. reported the inhibitory effects of SNPs on osteoclast differentiation and function, but only conducted in vitro experiments at corresponding concentrations [56]. In this study, we developed an Ag-incorporated implant using TiO_2_ nanotubes (TiO_2_-NTs) as the supporting matrix, enabling the stable and sustained release of ultra-low levels of Ag⁺ ions to satisfy the prolonged ionic requirements for osteoporotic bone regeneration. Our findings demonstrated that Ag@TiO_2_-NTs suppress osteoclastogenesis and bone resorption by downregulating the expression of genes and markers specific to osteoclast activity. Additionally, compared with Ti and TiO_2_-NTs, Ag@TiO_2_-NTs significantly enhanced the expression of osteogenesis-associated genes in MC3T3-E1 cells, confirming their role in promoting osteogenic differentiation. Furthermore, in vivo experiments revealed that, relative to the Ti and TiO_2_-NTs implant groups, Ag@TiO_2_-NTs markedly improved bone defect repair in osteoporotic rat models. Collectively, these results indicate that silver possesses therapeutic potential for osteoporosis treatment, and that Ag@TiO_2_-NTs with controlled, ultra-low-dose Ag⁺ release represent a promising strategy for regenerating osteoporotic bone defects.

**Figure 8:**
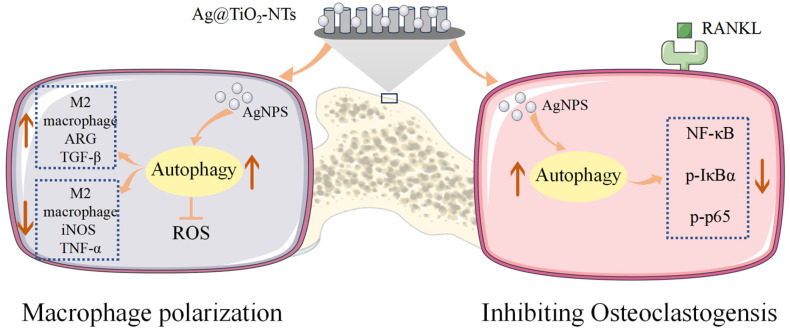
Illustration of Ag@TiO_2_-NTs’ modulatory role in macrophage polarization and inhibiting osteoclastogensis.

Autophagy is a ubiquitous cellular process that regulates cellular homeostasis and immunity and alleviates oxidative stress by removing reactive oxygen species (ROS) [57,58]. It plays a crucial role in maintaining cellular homeostasis in response to environmental stimuli. Previous studies have confirmed [44] that Ag@TiO_2_-NTs with the controlled release of ultra-low-dose Ag^+^ ions can regulate macrophage autophagy to clear ROS, promote M2 polarization, and create an osteogenic immune microenvironment rather than inhibiting the host response. This was also validated in our study, where compared to Ti and TiO_2_-NTs, Ag@TiO_2_-NTs significantly enhanced M2 polarization and the expression of osteogenic genes. Additionally, increased ROS in bone marrow-derived monocytes or macrophages can accelerate osteoclastogenesis through the activation of TNFα, TRAF6, and Rac1 [59]. Our data suggest that Ag@TiO_2_-NTs may inhibit RANKL-induced osteoclast differentiation by enhancing ROS clearance through macrophage autophagy. The NF-κB signaling pathway is one of the most important pathways for osteoclastogenesis. The transcription of target genes such as CTSK and cFos is stimulated by the phosphorylation of NF-κB signaling proteins. In RAW 264.7 cells, we observed that Ag@TiO_2_-NTs treatment significantly inhibited the phosphorylation of IκBα and p65. These results indicate that the potential molecular mechanisms through which Ag inhibits osteoclast formation may be identified. Indeed, our findings demonstrate that Ag@TiO_2_-NTs significantly enhance autophagy in macrophages under RANKL stimulation. In the context of osteoclastogenesis, autophagy has been shown to regulate the differentiation and function of osteoclast precursors [60]. Our study highlights that Ag@TiO_2_ induces autophagy in macrophages, which may contribute to the inhibition of osteoclast differentiation. The activation of autophagy was confirmed by the increased expression of LC3-II, a marker of autophagosome formation [61]. These findings suggest that Ag@TiO_2_-NTs promotes autophagy, which may serve as a protective mechanism to counteract the pro-inflammatory and osteoclastogenic signals induced by RANKL. The interplay between autophagy and the NF-κB signaling pathway is a critical aspect of this study. Autophagy has been shown to negatively regulate the NF-κB pathway by degrading IκBα and preventing its ubiquitination and subsequent degradation, thereby reducing the activation of NF-κB [62]. This mechanism may explain how Ag@TiO_2_-NTs suppresses osteoclastogenesis through the autophagy-mediated inhibition of the NF-κB pathway. Therefore, Ag@TiO_2_-NTs-induced bone regeneration in osteoporotic rats is considered to be associated with Ag^+^-mediated immune modulation, promoting osteogenesis and the autophagy-activated inhibition of RANKL-induced NF-κB signaling and leading to the suppression of osteoclast formation and resorption (Figure 8).

Unfortunately, there are some limitations in this study. First, the causes and progression of osteoporosis are complex, involving multiple factors. However, in our investigation, we only examined autophagy and NF-κB signaling pathways, without further exploring their relationship. Second, it remains unclear whether Ag directly acts on ROS through autophagy and NF-κB pathways or if there are certain molecular targets connected to autophagy and NF-κB. Additionally, we created and used a rat iliac bone osteoporotic bone defect model for the first time in this study, but some rats in the model experienced bone fractures. Further improvements may be needed for the creation of iliac bone defects and the implantation of materials. The study focuses primarily on an osteoporosis-related bone defect model, limiting its generalizability to other bone diseases such as fractures or infections. Short-term observations were made, but long-term studies are required to assess the sustained efficacy and safety of Ag@TiO_2_-NTs in clinical settings. The fabrication method used for Ag@TiO_2_-NTs nanotube arrays may not be scalable or suitable for the industrial production of implants, and the potential toxicological effects of AgNPs remain unexplored, despite showing no significant cytotoxicity at ultra-low concentrations in the study [63]. Moreover, the study uses rat models, which may not fully translate to human physiology, limiting its direct applicability to clinical practice; therefore, future studies should involve larger animal models to better replicate human conditions [64]. The study used a specific concentration of AgNPs, but the optimal concentration and delivery method for clinical applications require further investigation. Although this study offers valuable mechanistic insights into the effects of Ag@TiO_2_-NTs, further investigations are required to comprehensively elucidate the molecular and cellular interactions at play. The clinical use of AgNPs in implants requires rigorous testing to ensure their safety, biocompatibility, and absence of adverse immune responses, which may require extensive preclinical and clinical trials. The fabrication method may not be cost-effective or scalable for large-scale production, and a more reproducible manufacturing process is needed for clinical adoption. Moreover, the success of titanium implants in clinical settings depends on their integration with bone tissue, requiring further investigation into mechanical stability and long-term performance [65]. Individual patient variations, including health, immune responses, and bone quality, may affect the efficacy of Ag@TiO_2_-NTs implants, necessitating personalized medicine approaches to optimize outcomes. Additionally, patients with osteoporosis often have comorbidities that require medications, so potential interactions between AgNPs and other drugs must be evaluated. Overall, our work provides insights for future osteoporosis research and therapy.

## 5. Conclusions

In summary, Ag@TiO_2_-NTs with controlled release of ultra-low-dose AgNPs were fabricated and applied to regenerate bone defects in osteoporotic rats. The incorporation of AgNPs not only endowed TiO_2_-NTs with the ability to regulate macrophage polarization, but also inhibited osteoclast formation and function in vitro. Furthermore, in vivo, a satisfactory bone immune microenvironment was generated by the increased expression of wound-healing M2 macrophages around Ag@TiO_2_-NTs. Additionally, Ag@TiO_2_-NTs reduced osteoclast numbers and accelerated bone defect regeneration in osteoporotic rats by activating autophagy and inhibiting the RANKL-induced NF-κB pathway. Our study indicates that Ag@TiO_2_-NTs modified with ultra-low-dose AgNPs exhibit both immune modulation and excellent osteogenic capabilities for osteoporosis, making them a promising biomaterial for implant applications (Table 1).

## Figures and Tables

**Table 1:** A summary table of key results.

Section	Conclusions
Fabrication and Characterization	Ag@TiO_2_-NTs nanotubes are successfully fabricated with controlled Ag^+^ release and no significant cytotoxicity.
Osteo-immunoregulatory Ability In Vitro	Ag@TiO_2_-NTs regulates macrophage polarization toward anti-inflammatory M2 phenotype and enhances osteogenic activity.
Ag@TiO_2_-NTs Inhibited Osteoclastogenesis In Vitro	Ag@TiO_2_-NTs effectively inhibits osteoclastogenesis, reducing bone resorption.
Ag@TiO_2_-NTs Inhibited Osteoclastogenesis	Ag@TiO_2_-NTs inhibits osteoclastogenesis by promoting autophagy and suppressing the NF-κB pathway.
Ag@TiO_2_-NTs Stimulated Bone Regeneration	Ag@TiO_2_-NTs implants improve bone regeneration, reduce inflammation, and inhibit osteoclast activity in vivo.

## Data Availability

The raw data supporting the conclusions of this article will be made available by the corresponding author on request.

## Appendix A

**Table A1:** Primers used for real-time PCR.

Gene	Forward Primer Sequence (5′-3′)	Reverse Primer Sequence (5′-3′)
iNOS	GTTCTCAGCCCAACAATACAAGA	GTGGACGGGTCGATGTCAC
Arg1	CTCCAAGCCAAAGTCCTTAGAG	GGAGCTGTCATTAGGGACATCA
TGFa	CACTCTGGGTACGTGGGTG	CACAGGTGATAATGAGGACAGC
TGFb	CCACCTGCAAGACCATCGAC	CTGGCGAGCCTTAGTTTGGAC
ALP	GGCCTTTTACCTTTCACGGTG	TACGGCATTGTGGCTTCTCAA
OPG	ACCCAGAAACTGGTCATCAGC	CTGCAATACACACACTCATCACT
OCN	CTGAAAAGCCCACAGATACCAG	TGGAGAGGGTTGTTAGTGTGTC
RUNX2	ATGCTTCATTCGCCTCACAAA	GCACTCACTGACTCGGTTGG
NFATc1	GACCCGGAGTTCGACTTCG	TGACACTAGGGGACACATAACTG
c-Fos	CGGGTTTCAACGCCGACTA	TTGGCACTAGAGACGGACAGA
MMP9	CTGGACAGCCAGACACTAAAG	CTCGCGGCAAGTCTTCAGAG
TRAP	CACTCCCACCCTGAGATTTGT	CATCGTCTGCACGGTTCTG
Ctsk	GAAGAAGACTCACCAGAAGCAG	TCCAGGTTATGGGCAGAGATT
GAPDH	AGGTCGGTGTGAACGGATTTG	TGTAGACCATGTAGTTGAGGTCA
